# The Effects of Extraversion, Social Support on the Posttraumatic Stress Disorder and Posttraumatic Growth of Adolescent Survivors of the Wenchuan Earthquake

**DOI:** 10.1371/journal.pone.0121480

**Published:** 2015-03-27

**Authors:** Xuji Jia, Liuhua Ying, Xiao Zhou, Xinchun Wu, Chongde Lin

**Affiliations:** 1 Institute of Developmental Psychology, Beijing Normal University, Beijing, China; 2 Department of Psychology, Zhejiang Sci-Tech University, Hangzhou, China; Univ of Toledo, UNITED STATES

## Abstract

**Objective:**

The aim of this study was to examine the relationships among extraversion, social support, posttraumatic stress disorder and posttraumatic growth among adolescent survivors of the Wenchuan earthquake.

**Methods:**

Six hundred thirty-eight participants were selected from the survivors of the 2008 Wenchuan earthquake. Participants completed four main questionnaires, including the Extraversion Subscale, the Social Support Scale, the Child PTSD Symptom Scale, and the Posttraumatic Growth Inventory.

**Results:**

A bivariate correlation analysis revealed significant correlations among extraversion, social support, posttraumatic stress disorder and posttraumatic growth. Extraversion had significant indirect effects on posttraumatic stress disorder (β = −.037, *p* < .01) and posttraumatic growth (β = .077, *p* < .001) through social support. The results also indicated that extraversion had a significant direct effect on posttraumatic growth and a nonsignificant direct effect on posttraumatic stress disorder.

**Conclusions:**

Social support fully mediates the relationship between extraversion and posttraumatic stress disorder and partially mediates the relationship between extraversion and posttraumatic growth. Psychological interventions and care for survivors of the earthquake should include the various functions and sources of social support and how they serve to benefit individuals.

## Introduction

One of the most destructive natural disasters, the 2008 Wenchuan earthquake in China led to huge causalities and economic losses. According to official state statistics, it caused 68,712 deaths, 17,921 missing persons and direct economic losses of 8451 billion yuan. The earthquake also resulted in a range of negative and positive psychological consequences for survivors. Posttraumatic stress disorder (PTSD) is considered the most frequently reported negative psychological outcome following an earthquake [1–2]. As a positive psychological change, posttraumatic growth (PTG) following an earthquake has also been recently identified [3]. According to Tedeschi and his colleagues, PTG initially manifested in three broad categories, including perceived changes in the self, one’s sense of relationships with others, and one’s philosophy of life, and then expanded to five dimensions, including the ability to relate with others, new possibilities, personal strength, spiritual change, and an appreciation of life [4–5]. Research has revealed that PTG was widely reported following various types of traumatic life events, such as serious medical illnesses [6], accidents [7], the loss of a loved one [8], and natural disasters [9]. However, to our knowledge, very few studies have examined the PTSD and PTG of the Wenchuan earthquake survivors.

As a consequence of traumatic events, the association between PTG and PTSD has been an important research issue. There are three possible modes of the relationship: a positive relationship [5], a negative relationship [10–11], and no relationship [12–13]. Recently, a review demonstrated that most research on the relationship between PTG and PTSD among adults has consistently revealed a positive association between the two variables [14–15], and that PTG and PTSD share antecedents (i.e., seismic events) and processes [16]. Several studies conducted with children and adolescents using a longitudinal design also reported a positive relationship between the two variables [17–20]. To add to the existing knowledge on the relationship between PTG and PTSD among survivors of an earthquake, we examined whether there was a significant relationship between the two variables.

Based on the importance of PTG, theoreticians have conceptualized various models to explain the development of PTG [11]. Schaefer and Moos proposed a comprehensive model of posttraumatic growth to clarify the factors that contribute to the development of PTG. The model implies that both personal system factors (e.g., personality traits and prior crisis experience) and environmental resources (e.g., support from family and friends) combine to influence event-related factors during a life crisis or a transition period. They influence cognitive appraisal processes and coping responses, which, in turn, influence posttraumatic outcomes [21].

As one of the important personal system factors, personality traits play an important role in the development of PTG and PTSD. The major personality traits, particularly neuroticism and extraversion in the Five Factor Model [22], may in part account for differences in reactions to exposure to traumatic events [23–24]. Associations have consistently indicated that neuroticism is negatively related to PTG and positively related to PTSD [25–27]. However, evidence indicates that the relationships between extraversion and PTG, and PTSD are less consistent [28]. For example, several studies have found that extraversion is one of the best predictors of PTG and negatively related to PTSD [29–31]. However, other researchers found no relationship between extraversion and PTSD [27, 32] or between extraversion and PTG [33]. Thus, the effects of extraversion in the domains of PTSD and PTG have not been extensively researched. In the present study, we selected extraversion as the personality trait of interest and examined the effect of extraversion on PTSD and PTG in adolescent survivors of the Wenchuan earthquake.

Social support, an environmental factor, is often considered one of the most important precursors in the development of PTG and PTSD. Social support is in itself a multidimensional concept comprising structural social support and functional social support [34–35]. Structural social support refers to the quantity and quality of available relationships or social roles, such as the frequency of an individual’s contact with various network members and the density and complexity of relationships among network members. Functional social support refers to the degree to which an individual believes that his/her needs for companionship, intimacy, and esteem are fulfilled [36]. Most researchers agree that the structural and functional aspects of social support are different phenomena and should be studied separately [34]. Studies have identified various magnitude of relationships between structural social support and PTG, and these differences are to some extent based on the different support sources involved (e.g., family support, teacher support, and peer support)[37–39]. Two meta-analysis studies also revealed that structural social support was the strongest predictor of PTSD, yielding effect sizes of. 40 and. 28 [40, 41]. An individual’s perception of functional social support is as important as his/her perception of structural social support during traumatic events. Based on this view, researchers have attempted to explore the role of different degrees of functional social support in the development of PTSD and PTG. For example, some studies found that more functional support predicted a greater experience of PTG and PTSD in victims of cancer [42,43] and that functional social support was related to the severity of PTSD in victims of violent crime and sexual assault[44,45]. However, to our knowledge, little research has focused on functional social support in relation to PTG and PTSD as a result of earthquakes. In the present study, we explored the role of functional social support in PTSD and PTG in adolescent survivors of the Wenchuan earthquake.

In addition, research has reported that extraversion is related to social support. Previous studies on the relationship between extraversion and structural social support revealed that more highly extroverted individuals were likely to have larger social support networks [46] and more frequent contact with others [47] and to report a higher likelihood of seeking social support [48]. Similarly, studies on the relationship between extraversion and functional social support have also indicated significant correlations with scores on the social provisions scale [49–50].

Moreover, researchers have identified some mediating variables (e.g., a coping strategy, social support) in the relationship between extraversion and PTSD, PTG [29]. For instance, individuals scoring high on extraversion were expected to better engage with social support following trauma exposure, which, in turn, would reduce PTSD symptoms [27, 51]; extraversion had a significant positive relationship with social support, which, in turn, mediated the effect of extraversion on both sense-making and benefit-finding [52].

Based on the literature reviewed, previous studies on the links among the four variables described above have been fruitful in establishing the independent contribution of each variable. However, the mechanisms by which extraversion and social support jointly influence PTSD and PTG were unclear. In the present study, based on the framework of Schaefer and Moos’s model, we simultaneously examined the effects of extraversion (a personal system factor) and social support (an environmental factor) on PTSD and PTG within the same sample.

**Fig 1:**
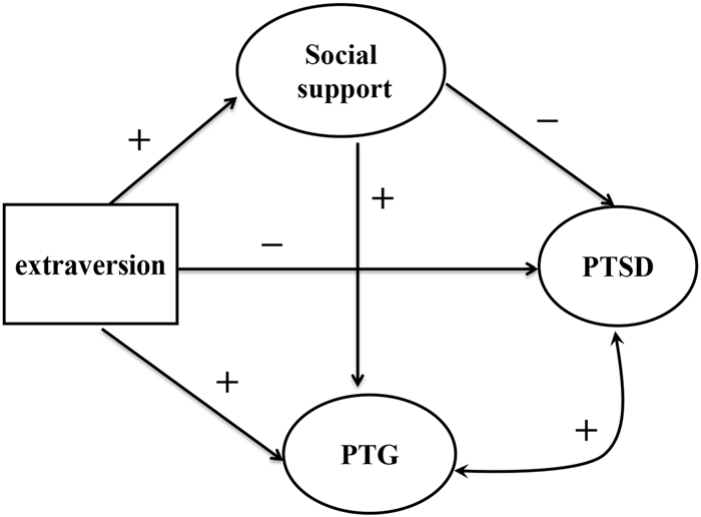
The hypothesized model of posttraumatic growth. This figure describes the hypothesized paths of extraversion, social support, posttraumatic stress disorder, and posttraumatic growth. PTSD = posttraumatic stress disorder; PTG = posttraumatic growth; arrows imply the directionality in the model, “+” indicates a positive prediction, “—” indicates a negative prediction.

Therefore, the main purpose of the present study was to examine the pathways between extraversion and social support in relation to PTSD and PTG among adolescent survivors of the Wenchuan earthquake. We hypothesized that (a) extraversion would be positively related to social support; (b) social support would be negatively related to PTSD and positively related to PTG; (c) extraversion would be negatively related to PTSD and positively related to PTG; (d) PTSD would be positively related to PTG; and (e) social support would mediate the relationship between extraversion and PTSD, and PTG. We used a structural equation model (SEM) to test the hypothesized model among adolescent survivors of the Wenchuan earthquake. The model is presented in Fig. 1.

## Methods

### Participants and procedures

This study was part of a larger investigation of psychological adjustment among survivors of the Wenchuan earthquake in China. The sample for the current study consisted of 638 middle school students in the Wenchuan area. Of the 638 returned surveys, 7 were excluded because the respondent did not complete the majority of the survey. Therefore, 631 surveys were used for further analyses. Of these surveys, 241 (38.19%) were completed by male participants and 390 (61.81%) were completed by female participants; 196 (31.06%) were students in the seventh grade, 211 (33.44%) in the eighth grade, 84 (13.31%) in the tenth grade, and 140 (22.19%) in the eleventh grade. The mean age was 15.13 years (*SD* = 1.75). With respect to ethnicity, 142 (22.50%) belonged to the Han ethnic group, 139 (22.03%) belonged to the Tibetan ethnic group, 319 (50.56%) belonged to the local Qiang people, and 31 (4.91%) belonged to other minor ethnic categories.

This project was approved by the local education authorities and the Research Ethics Committee of Beijing Normal University. Written informed consent was obtained from the school principals and classroom teachers. In China, research projects that are approved by local education authorities, such as county departments of education and the school administrators, and that are deemed to provide a service to the students do not require parental consent. The Research Ethics Committee of Beijing Normal University specifically approved this method of informed consent for school principals and teachers in lieu of parental consent. Before the formal surveys were administered, written consent was obtained from the school administrators and teachers along with oral approval from the participants. Two trained research assistants administered the surveys in these schools 12 months after the Wenchuan earthquake. We informed participants that completing the surveys was voluntary, that their anonymity would be ensured and that they had a right to withdraw from the study at any time. Participants with extremely high PTSD symptoms were referred to clinical psychologists in Chengdu and Beijing for further treatment.

### Measures

#### Personality

The Five-Factor Personality Questionnaire [53] was used to assess extraversion. The questionnaire was adapted for middle school students in China based on Costa and McCrae’s five-factor model of personality. It includes five subscales: extraversion, neuroticism, openness, agreeableness, and conscientiousness. Participants responded on a 5-point Likert-type scale, with responses including *strongly disagree*, *disagree*, *neutral*, *agree*, *and strongly agree*. The questionnaire had satisfactory reliability and validity [53] and has been used to assess students’ personalities in various studies [54–55]. The extraversion subscale with 11 items had good internal consistency in our sample, with a Cronbach’s alpha of. 87.

#### Social Support

Social support was measured with a revised social support scale based on Furman and Buhrmester's Network of Relationships Inventory [56–57]. This revised scale includes five provisions of social support: emotional support, instrumental support, companionship, intimacy and enhancement of worth. This scale consists of 20 items to assess functional social support qualities. For each item, participants indicated the extent to which they have experienced the type of support on a 5-point Likert-type scale ranging from 0 (‘‘not at all”) to 4 (‘‘always”) since the occurrence of the Wenchuan earthquake until the present. The scale has been administered to samples of survivors to assess functional social support after an earthquake with high reliability and construct validity [58]. In the current study, the Cronbach’s alpha coefficients of the total scale and the five subscales ranged from. 88 to. 95.

#### PTSD

PTSD was evaluated using the Child PTSD Symptom Scale (CPSS), which has 17 items [59]. These items were combined into three subscales namely, intrusion (5 items), avoidance (7 items), and hyperarousal (5 items). Children rated the frequency of experiencing the given symptoms over the previous 2 weeks on a 4-point Likert scale ranging from 0 (“not at all”) to 3 (“almost always”). The psychometric properties of the scale have been reported to be adequate [59]. The scale was adapted to Chinese with satisfactory psychometric properties [60]. For the present study, the Cronbach’s alpha values were. 89 for the total scale,. 82 for the intrusion subscale,. 73 for the avoidance subscale, and. 77 for the hyperarousal subscale.

#### PTG

PTG was assessed using the revised Posttraumatic Growth Inventory (PTGI). The original PTGI developed by Tedeschi and Calhoun consisted of 21 items covering five subscales [4]: personal strength, new possibilities, the ability to relate to others, appreciation of life, and spiritual change. The modified PTGI was composed of 22 items with responses ranging from 0 to 5 (0 = “no change” to 5 = “great change”). Confirmatory factor analysis supported a three-factor model, and the degrees of model fit were as follows: χ^2^/df = 2.35, CFI = .93, RMSEA = .07, demonstrating that the revised version has good reliability and construct validity in studies of PTG [58, 61]. The revised scale consisted of three subscales: perceived changes in the self (9 items), the sense of relationship with others (7 items), and changed philosophy of life (6 items). In the present study, the Cronbach’s alpha values were. 93 for the total scale,. 88 for the perceived changes in the self subscale,. 86 for the sense of relationship with others subscale, and. 72 for the changed philosophy of life subscale.

## Results

### Descriptive statistics

Based on the DSM-IV [62], participants were identified as having full PTSD according to the following criteria: (a) one or more items on the intrusion subscale scored 2 or 3; (b) three or more items on the avoidance subscale scored 2 or 3; and (c) two or more items on the hyperarousal subscale scored 2 or 3. According to these criteria, the prevalence rate of probable PTSD was 28.37%. In addition,according to the cutoff points for PTG in Tang's study [63], average mean scores above 3 on each item of the PTGI were indicative of moderate levels of posttraumatic growth. Therefore, scores of 66 and above were considered to indicate a moderate level of PTG in the present study. Approximately 62.76% of participants reported a moderate level of PTG.

The bivariate correlations among the main variables are presented in Table 1. As shown in Table 1, extraversion and social support were positively related (*r* = .27, *p* <.001). Both extraversion and social support had significantly negative correlations with PTSD (*r* = -.10, *p* <.05; *r* = -.14, *p* <.01, respectively) but significantly positive correlations with PTG (*r* = .32, *p* <.001; *r* = .35, *p* <.001, respectively). PTSD was positively associated with PTG (*r* = .10, *p* <.05). Sex (0 = female, 1 = male) was significantly correlated with social support, PTSD and PTG, but its correlation with extraversion was non-significant. Grade and age were significantly correlated with other study variables, except for PTG. Ethnicity did not correlate with any main study variables. Therefore, we controlled for sex, grade and age in the subsequent analysis.

**Table 1:** The correlations, means, and standard deviations among the main study variables.

Variables	Correlations								*Mean* ± *SD*
	1	2	3	4	5	6	7	8	
**1. Sex**	-								—
**2. Grade**	-.15***	-							—
**3. Ethnicity**	-.10*	.14***	-						—
**4. Age**	-.10*	.89***	.10*	-					15.13±1.75
**5. Extraversion**	.01	-.12**	-.02	-.12**	-				25.92±8.47
**6. Social support**	-.12**	-.13**	.07	-.15***	.27***	-			49.87±18.37
**7. PTSD**	-.20***	.15***	.01	-.18***	-.10*	-.14***	-		15.84±8.49
**8. PTG**	-.10**	.01	.02	-.01	.32***	.35***	.10*	-	68.29±20.06

PTSD = posttraumatic stress disorder. PTG = posttraumatic growth.

*p <. 05

**p <. 01

***p <. 001.

### Structural equation modeling

Structural equation modeling (SEM) was employed to test our hypothesized model using Mplus software (Version 7). In our hypothesized model, PTG was indicated by three dimensions: perceived changes in the self, the sense of having relationships with others and changed philosophy of life. PTSD was indicated by intrusion, avoidance and hyperarousal. Social support was indicated by five provisions of social support: emotional support, instrumental support, companionship, intimacy and enhancement of worth. Extraversion was measured by only one indicator.

In Mplus, the model parameters were estimated using maximum likelihood (ML). The degree of model fit was assessed using the following fit indices [64]: the chi-square statistic, the root mean squared error of approximation (RMSEA), the comparative fit index (CFI), and the standardized root mean square residual (SRMR). The guidelines for an acceptable fit included a non-significant χ^2^ value, a RMSEA value less than. 10, a CFI value greater than. 90, and an SRMR value less than. 10. Because bias-corrected bootstrapping has greater statistical power to detect mediation effects than other forms of mediation analyses [65], we used a bias-corrected bootstrap estimation with a 95% confidence interval to determine the significance of the indirect effects. We created 5000 bootstrap samples using the original dataset.

We first tested the hypothesized model. Preliminary analysis indicated that the data met the assumption of normality based on the criteria recommended by Weston and Gore [64]. SEM analyses revealed that all coefficients for the direct and indirect paths in the hypothesized model were significant, except for the direct path from extraversion to PTSD (β = -.017, *p* = .165).

Then, we compared our hypothesized model with three alternative models to examine whether the relationships between extraversion and the dependent variables (PTSD and PTG) were partially or fully mediated. Alternative model 1 constrained the path from extraversion to PTSD to zero. Alternative model 2 constrained the path from extraversion to PTG to zero. Alternative model 3 simultaneously constrained the above two paths to zero. The model fit indices of these models are presented in Table 2.

**Table 2:** Fit indices of the hypothesized and alternative models.

Model	χ^2^	*df*	RMSEA [90% Confidence Interval]	CFI	SRMR
**Hypothesized model**	210.642***	76	.053[.045,. 062]	.975	.041
**Alternative model 1**	212.822***	77	.053 [.044,. 061]	.974	.041
**Alternative model 2**	248.870***	77	.059 [.051,. 068]	.967	.051
**Alternative model 3**	254.448***	78	.060[.052,. 068]	.967	.051

***p <. 001.

The result of the chi-square difference test between the hypothesized model and alternative model 1 was non-significant, Δχ^2^(1) = 2.180, *p* >. 05, suggesting that constraining the path from extraversion to PTSD was necessary based on the principle of parsimony and that alternative model 1 was better fitted to the data. The results of chi-square difference tests between the hypothesized model and alternative models 2 and 3 were significant, Δχ^22^(1) = 38.228, *p* <. 001 and Δχ^22^(2) = 43.806, *p* <. 001, respectively, suggesting that constraining those paths significantly reduced the model fit and that the hypothesized model should be retained. From the results of the fit indices of the RMSEA, CFI and SRMR in four models, we selected alternative model 1 as our final model.

**Fig 2:**
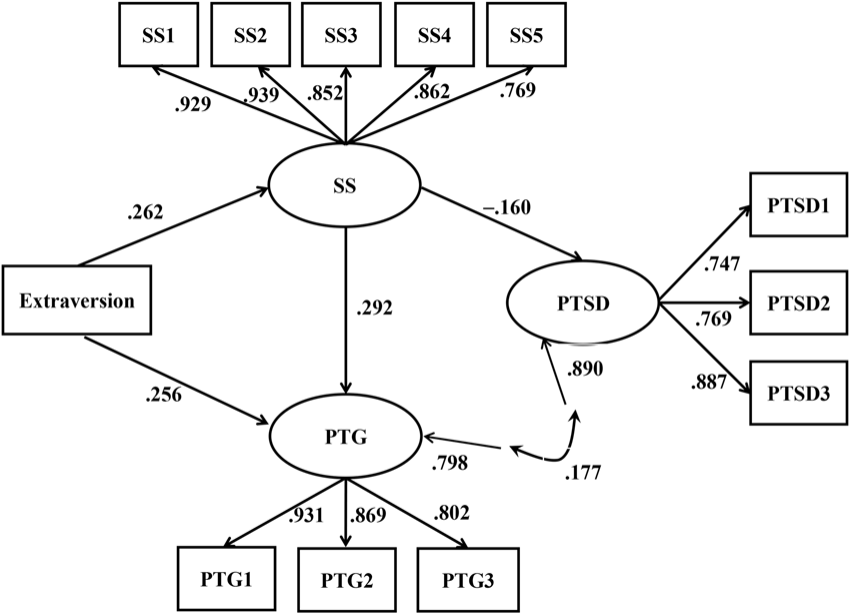
The structural equation modeling results of the final model. This figure reveals the standardized path coefficients in the final model from Mplus after controlling for sex, grade and age. All path values, *ps* <. 001. Fit indices of the model: χ^2^ (77, N = 631) = 212.822, *p* <. 001; root mean square error of approximation [90% confidence interval] = .053 [.044, .061]; comparative fit index = .974; standardized root mean square residual = .041. SS = social support; SS1 = emotional support; SS2 = instrumental support; SS3 = companionship; SS4 = intimacy; SS5 = enhancement of worth. PTSD = posttraumatic stress disorder; PTSD1 = intrusion; PTSD2 = avoidance; PTSD3 = hyperarousal. PTG = posttraumatic growth; PTG1 = perceived changes in the self; PTG2 = sense of relationships with others; PTG3 = changed philosophy of life.

The parameter estimates of the final model are depicted in Fig. 2. The results of the bootstrap procedure (see Table 3) revealed that extraversion had significant indirect effects on both PTG (β = .077, *p* <.001) and PTSD (β = -.037, *p* <.01) via social support. Moreover, the direct effect of extraversion on PTG was also significant (β = .256, *p* <.001).

**Table 3:** Bootstrap analysis of the magnitude of the effects in the final model.

Independent variable	Mediator variable	Dependent variable	B-Standardized estimate	SE-Standardized error	95% confidence interval
**Extraversion**		SS	.262***	.040	[.198,. 325]
**Extraversion**		PTG	.256***	.042	[.191,. 325]
**SS**		PTG	.292***	.043	[.225,. 365]
**SS**		PTSD	-.160***	.045	[-.238, -.078]
**Extraversion**	SS	PTG	.077***	.016	[.051,. 103]
**Extraversion**	SS	PTSD	-.037**	.014	[-.060, -.015]

SS = social support. PTSD = posttraumatic stress disorder. PTG = posttraumatic growth.

**p <. 01

***p <. 001.

## Discussion

The goal of this study was to test a comprehensive model including types of social support as mediating variables in the relationship between extraversion and PTSD and PTG among adolescent survivors of the Wenchuan earthquake. Although previous research has documented links among subsets of extraversion, social support, PTSD and PTG [20, 30, 42, 51, 66], no previous work has conceptualized the relationships among these four variables as a group to test the proposed meditational effect. We compared the hypothesized model with three alternative models and selected the model with the best fit to the data. The findings of this study supported and extended Schaefer and Moos’s model in survivors of earthquakes by suggesting more complicated relationships among the four variables.

The results indicated that social support was negatively associated with PTSD but positively associated with PTG. In our study, social support is defined as the degree to which an individual believes that his/her needs are fulfilled and refers to its functional rather than structural aspects. Previous studies have shown that structural social support had a negative effect on PTSD and a positive effect on PTG [38, 67]. The current study further demonstrated the opposing effects of functional social support on PTSD and PTG. Cognitive processing theory provided an explanation for the important role of social support in the development of PTSD and PTG [5, 68]. According to this theory, by offer different provisions of social support (e.g., opportunities for self-disclosure and offering advice or new perspectives), social support may facilitate cognitive processes and the utilization of coping strategies that may contribute to reducing symptoms of PTSD and identifying positive meaning to access personal growth [42].

The results also indicated a positive association between extraversion and social support. This finding was in line with previous studies reporting that extroverted individuals exhibit higher levels of social support [48–49]. Because they are more sociable and enjoy being in the company of others, extroverts are more likely to make more friends and obtain more emotional support, to share their experiences or secrets with others (intimacy support) and to ask for tangible help in times of stress (instrumental support)[49, 51]. Moreover, extroverts tend to involve more peers in common recreational activities (companionship support) and affirm their competence or value by comparing themselves with others (enhancement of worth). Therefore, it is plausible to find a positive association.

More importantly, as predicted, extraversion had significant indirect effects on PTSD and PTG via social support, which is consistent with previous research reporting a mediating effect of social support [51–52]. This finding suggests that social support could partially mediate the relationship between extraversion and PTG and fully mediates the relationship between extraversion and PTSD. This implies that extroverted individuals may foster both an individual’s ability to create a supportive environment and mobilize different support resources during difficult experiences, which could not only contribute to alleviating the symptoms of PTSD but also help individuals find benefits in difficult life experiences [49, 52]. It is notable that the mediating effects were very small in the present study and may need to be further validated.

A significant direct effect of extraversion on PTG was observed, which was consistent with previous studies reporting that extraversion was positively correlated with PTG [25, 51]. The direct effect of extraversion on PTG might suggest that extraversion in and of itself is beneficial to individual growth. One possible explanation is that extroverts are more likely to express their emotions and expose themselves to others, which may promote PTG in interpersonal interactions. Another possible explanation is that extroverted persons have more optimistic traits. Certain findings indicated that optimism independently contributed to PTG [69]. Optimistic individuals tend to focus on the most important matters and reject unachievable goals that are inconsistent with reality, which could lead to PTG. However, we should note that optimism may serve as a confounding factor in the relationship between extraversion and PTG, which would need to be further validated in future research.

Moreover, there was a negative correlation between extraversion and PTSD in the correlation analysis. However, we failed to identify this association in our final model, which contradicted our hypothesis. This finding supported the results of Schnurr et al.’s study [32]. It may indicate a different pathway for the effects of extraversion on PTSD and PTG.

In addition, the results supported the notion that PTSD and PTG are positively related. We observed this positive relationship in the results of the correlation and SEM analyses. This result was consistent with previous findings [5, 70] and supported the view that PTG and PTSD may co-exist simultaneously in individuals.

The findings of this study should be interpreted with caution. First, the current study was a cross-sectional design, which limited its ability to draw causal inferences regarding the observed relationships. Thus, future studies will need to adopt longitudinal designs to fully disentangle the underlying causality among the variables. Second, the sample consisted of middle school students who experienced the Wenchuan earthquake. The data were collected 12 months after the Wenchuan earthquake. Thus, the results of the present study should be cautiously generalized to other participant groups and time periods (e.g., 6 months after an earthquake). Third, extraversion, social support, PTSD and PTG were all assessed using single measures, which may have resulted in potential biases. Replicating this study using multiple measures of each construct could provide more information on the constructs and the relationships between them.

Despite these limitations, this study contributes to the literature by exploring the mediating role of social support in the relationship between extraversion and PTG, PTSD. Our findings have certain implications for clinical practice. Because extroverted individuals may report greater social support, which fosters PTG and reduces symptoms of PTSD, it is important for clinicians to teach survivors of an earthquake how to mobilize social support resources to cope with traumatic experiences. For example, a psychosocial group intervention should be developed to provide support and the opportunity to discuss earthquake-related concerns and thoughts. There is evidence that such a group intervention may decrease the symptoms of PTSD [71] and increase the perception of PTG [72]. In addition, the current findings reveal that extraversion has a direct effect on PTG. Therefore, interventions to promote PTG can target personal resources, such as extraversion. Although personality traits such as extraversion generally refer to stable and enduring dispositions that may be relatively resistant to change over a brief period of time, interventions can nevertheless be aimed toward promoting those attitudes, expectancies, and behaviors that are characteristic of this particular personality trait [29]. Indeed, recent research indicates that there is a relationship between extraversion and finding positive meanings and that simply adopting extroverted behavior can spur positive feelings and increase one’s sense of wellbeing [73]. By these avenues, psychological interventions may help survivors to adjust to suffering and find positive meanings in surviving an earthquake.
